# Psoriasis and Metabolic Syndrome: Comorbidities and Environmental and Therapeutic Implications

**DOI:** 10.7759/cureus.6369

**Published:** 2019-12-12

**Authors:** Cesar Peralta, Pousette Hamid, Humera Batool, Zeina Al Achkar, Pierre Maximus

**Affiliations:** 1 Internal Medicine, California Institute of Behavioral Neuroscience and Psychology, Fairfield, USA; 2 Neurology, California Institute of Behavioral Neurosciences and Psychology, Fairfield, USA; 3 Internal Medicine, California Institute of Behavorial Neurosciences and Psychology, Fairfield, USA; 4 Internal Medicine, California Institute of Behavioral Neuroscience and Pshycology, Fairfield, USA

**Keywords:** psoriasis and metabolic syndrome, psoriasis and comorbilities, psoriasis and diet, lifestyle modification and psoriatic diseases

## Abstract

Psoriasis (PS) is an incessant, fiery skin sickness characterized by erythematous plaques with thick silvery scales, white or red patches of the skin, which encompasses several immunological, biomolecular, genetic, and environmental factors that may lead to further development of metabolic syndrome (MS) and vice versa. Metabolic syndrome is composed of multiple components (high blood pressure, abdominal obesity, glucose intolerance, and dyslipidemia) of risk factors that arise primarily from insulin resistance, mostly mediated by inflammatory cytokines, such as tumor necrosis factor alpha (TNF-α), interleukin-6 (IL-6) together with leptin and adiponectin, which are molecules also found in PS. The incidence, severity, and poor prognosis of the psoriatic diseases could be influenced by cardiometabolic diseases, which are controllable or preventable with intense lifestyle modification such as diet, exercise, and weight control. We performed a far-reaching writing search of different databases as part of this review; 47 investigations were regarded as important based on our search. Fasting, proper weight management, and special diet modifications seemed to have a positive impact on the management of PS. This review agrees with previous literature that nutritionists and specialists of preventive medicine should play a central role in the evaluation and management of psoriatic patients. We recommended that the management of this disease should focus on the environmental factors first instead of the genetic and immunologic pathways.

## Introduction and background

Psoriasis (PS) is an incessant, fiery skin sickness characterized by erythematous plaques with thick silvery scales, white or red patches of the skin, which appear regularly in the scalp, knee, elbows, and intergluteal zones. The disease involves hyperproliferation of the keratinocytes in the epidermis with an increase in the epidermal cell turnover rate. The disease is estimated to affect two to three percent of the population [1]. Its type and presentation vary among different populations [2]. Other types, such as inverse, nail, pustular, and erythrodermic, can also occur. Hereditary, environmental, and immunologic elements seem to assume an essential job in the advancement, seriousness, and PS's relationship with metabolic syndrome (MS) [3]. MS is defined by multiple components (high blood pressure, abdominal obesity, glucose intolerance, and dyslipidemia) of risk factors that arise primarily from insulin resistance mostly mediated by inflammatory cytokines, such as tumor necrosis factor alpha (TNF-α), interleukin-6 (IL-6) together with leptin and adiponectin, which are molecules also found in PS [4]. MS by itself could cause an increased risk for cardiovascular diseases (CVD) and premature mortality.

Several studies have revealed a strong association between MS and psoriatic diseases, regardless of PS severity or casualty [1-2]. Furthermore, other studies are establishing the association of the increase of body mass index (BMI), hip and waist circumference, and insulin concentration (which are the main components in MS) with the severity of PS [3]. However, reports also suggest that PS is a significant independent risk factor for cardiometabolic diseases and insulin resistance, regardless of BMI. Also, a systemic inflammatory state known as the “psoriatic march” is believed to be the cause behind the interplay of inflammatory mediators, such as cytokines and adipokines, that lead to insulin resistance, atherosclerosis, and cardiovascular diseases (CVD), such as acute coronary syndromes and cerebral infarction [4, 5]. Factors related to cardiovascular events, such as smoking, alcohol, and physical inactivity, are also prevalent among psoriatic patients. These could be potential causes of comorbidities [5,6]. PS, MS, hypertension, and aging share in common an increase in C-reactive protein (CRP), which may implicate an extra burden for increased cardiovascular risk [7].

In this review, we have attempted to clarify and emphasize how important it is to treat the comorbidities associated with psoriatic diseases. Since these comorbidities make the treatment of PS more difficult, they are associated with a higher healthcare resource utilization and costs among patients with PS and unfavorable prognosis of the disease [8]. Additionally, we provide a clear picture of the assessment and correlation of MS and, therefore, the severity of PS and the impact of the way of life adjustment in response to systemic and biological treatment of psoriatic conditions.

## Review

Metabolic syndrome and psoriasis

There are several definitions for the MS. The most broadly used one was put out by the National Cholesterol Education Program (NCEP) and the Adult Treatment Panel guidelines (ATP III) in 2001, which was revised in 2005 in a statement from the American Heart Association (AHA)/National Heart, Lung, and Blood Institute (NHLBI) [9]. Current ATP III criteria define MS as the presence of any three of the following five traits: 1) abdominal obesity, defined as a waist circumference of ≥102 in men and ≥88 cm in women; 2) serum triglycerides of ≥150 mg/dL or medication treatment for raised triglycerides; 3) high-density lipoprotein (HDL) cholesterol of <40 mg/dL in males and <50 mg/ dL in females or treatment for low serum levels of HDL cholesterol; 4) blood pressure level of ≥130/85 mmHg or treatment for hypertension; fasting glycemic levels of ≥100 mg/dL or treatment for hyperglycemia

Previous studies have observed that people with PS have higher odds of having MS, reporting a higher prevalence and incidence of obesity [9-12]. Ferdinando et al. showed a prevalence of MS in PS patients (49.4%) when compared to control group [35.0%, p = 0.04, odds ratio (OR) = 1.8, 95% confidence interval (CI) = 1.02 -3.23]. This study also showed a higher systolic blood pressure, BMI, glucose, waist circumference, more angina pectoris, and lower HDL values in PS patients than control, which can contribute as an additional cause for CVD as HDL has multiple anti-atherogenic properties. Also, these patients were older, and the prevalence of tobacco smoking was more common in psoriatic patients with MS (p = <0.05) [9]. Additionally, the presence of hypertension, type 2 diabetes (DM2), and MS are associated with an increase of severity and a larger duration of the disease. Psoriatic patients tend to have a higher intima-media thickness (mean: 0.61 mm ±0.01 vs 0.37 mm ±0.01) than the control groups [10].

Salihbegovic et al state that, although other studies have found no correlation between MS and the worsening of PS, their study demonstrated a strong significance between the two conditions as measured by the Psoriasis Area and Severity Index (PASI) score and MS (r = 0.35, p = 0.0001) [11]. Abdominal obesity, which is one of the most important components in MS, is strongly associated with the severity of psoriatic diseases, especially in young women compared with older females, thus leading to the conclusion that a decrease in waist circumference may improve symptoms of PS. Therefore, insulin resistance has been seen to block keratinocyte differentiation and can be involved in the pathogenesis of PS, MS, and CVDs [12]. It is vital to mention that age and sex variations in the risk of MS in patients with PS can exist. There are some findings that PS was associated with three to eight times higher odds of MS at age 30 years (95% CI: 1.5-9.7) with a decreasing OR with increasing age in women. In men, PS was associated with a stable, 1.35-times higher odds of MS (95 CI: 1.1-1.6) at all ages [12]. As we mentioned earlier, some studies have established that, although there is a significant connection between the two diseases, no relationship exists in terms of BMI, waist size, age, gender, and severity of PS. This lack of connection in previous studies could be due to the small number of participants [13].

The exact underlying pathway linking the two diseases is complex and not fully understood. However, in several studies, it has been observed that in both diseases inflammatory and anti-inflammatory markers produce oxidative damage to nucleic acids (DNA/RNA damage; 8-hydroxy-2′-deoxyguanosine, 8-hydroxyguanosine, and 8-hydroxyguanine). Adipokines, secreted by adipose tissue, increases insulin sensitivity and display antiatherogenic and anti-inflammatory effects. Adiponectin causes insulin-sensitizing effect by binding to the receptors AdipoR1 and AdipoR2, prompting activation of adenosine monophosphate dependent kinase (AMPK), peroxisome proliferator-activated receptor alpha (PPAR-α), and apparently other yet obscure flagging pathways. Weight reduction essentially raises plasma adiponectin levels. Insulin resistance, dyslipidemia, and atherosclerosis are related to a decrease in adiponectin levels. Leptin is another major adipokine leptin. Its major role is to hinder hunger, fire up thermogenesis, boost fatty acid oxidation, improve hyperglycemia, and diminish body weight and fat. The other is resistin, which can trigger a proinflammatory state by regulating various biological processes, thus contributing to inflammatory diseases and CRP, which is one of the most used makers worldwide for inflammation. Those studies revealed significantly higher CRP (p = <0.001), lipoprotein-associated phospholipase A2 (Lp-PLA2) (p = <0.001), leptin (p = <0.01), and resistin (p = <0.01) serum levels in patients with PS. Significantly higher levels of CRP (p = <0.01), Lp-PLA2 (p = <0.001), leptin (p = <0.01), and resistin (p = <0.05) were found in the patients with MS and PS compared to the controls with MS without PS. The level of adiponectin was significantly lower (p = <0.01) in the patients with MS. Surprisingly, a higher level of Lp-PLA2 (p = <0.001) was found in the group of patients without MS with PS compared to the controls without MS and PS, which correlates with the presence of subclinical atherosclerosis (cardiovascular risk) in psoriatic patients [14, 15]. Furthermore, in other studies, the levels of leptin and adiponectin or excess of weight gain did not correlate with the severity of PS. This could be attributed to the use of anti-inflammatory medications in the patients of the study. These medications can lower leptin values and increase adiponectin levels in psoriatic patients [16].

Psoriasis and comorbidities

Comorbidities in PS is a controversial issue due to the existence of several hypotheses stating PS is a direct cause of MS and other diseases or vice versa. Cytokine gene polymorphisms influence has been linked with the development of diseases like diabetes and psoriatic arthritis (PsA) independent of MS and the severity of PS [17]. Abnormalities of fatty acids profiles may prompt the advancement of metabolic disturbances and increase disease severity (high PASI score associated with low levels of Docosahexaenoic acid (DHA), n-3 polyunsaturated fatty acids (n-3 PUFA) (p = 0.044 and p = 0.048, respectively) (high DHA and n-3 PUFA levels linked with low inflammation and cardiovascular risk) and high percent of monounsaturated fatty acids (MUFA) (p = 0.024) in the non-obese psoriatic group. The saturated fatty acids (SFA)/unsaturated fatty acids (UFA) ratio increased with the duration of the disease (p = 0.03) in all psoriatic patients, suggesting an increased risk for cardio-metabolic diseases [18]. Different pathologies like non-alcoholic fatty liver disease (NAFLD) and others associated with high serum levels of triglycerides and uric acid, may play a role in the pathogenesis of MS and severity of PS but this could vary according to the time of the onset of PS [19]. Rising sphingosine-1-phosphate levels, which comes from ceramide (CER) breakup, and decrease in CER serum levels in psoriatic patients, could be one of the factors for the progression of skin lesions. These low ceramide levels point the way to an enemy of apoptotic and pro-proliferative epidermal condition, and thus to over-expansion of keratinocytes and the flourishing of skin lesions. Studies have exhibited a profoundly noteworthy and affirmative relationship between the rate decrease of CER and PASI score in PS while different research has exposed contradictory findings. However, higher degrees of CER was found in PsA patients contrasted with PS without arthritis. This proposes a conceivable job of CER additionally in psoriatic joint disease, which for the most part, are the patients with increasingly extreme sickness and typically have a tendency to develop more comorbidities such as DM2, hypertension, rheumatoid arthritis, and ankylosing spondylitis when compared to psoriatic patients without arthritis. [20]. Surprisingly, the history of gallstones in women may be an important risk factor in the development of PS and PsA (multivariate-adjusted RR = 2.20, 95% CI: 1.56-3.10) and concomitant PsA (multivariate-adjusted RR = 4.41, 95% CI: 2.70-7.18) independent of obesity [21]. Increased erythrocyte sedimentation rate, CPR, aging, and prediabetes (HbA1c: 5.7-6.4%) in patients with PS enhance the accumulation of advanced glycation end products (AGEs), contributing to accelerate the atherosclerosis and increased cardiovascular risk-based in the assessment of skin autofluorescence (SAF) as a measure of AGE accumulation (p = <0.05) [22].

Hypertension is a well-known end-organ damage disease and a cardiovascular risk factor that contributes to the development of myocardial ischemia and infarction, stroke, and cardiovascular death. Several studies have reported an association between PS, hypertension, and metabolic syndrome, especially because of the increasing levels of CPR or chronic inflammation that three pathologies shared in common [7]. Thus, PS incidence could be influenced or increase by hypertension in both females and males of <65 years [hazard ratio (HR): 1.54, 95% CI 1.47-1.61, p = <0.001) and the use of medications like calcium channel blockers (CCB) and thiazides (HR: 1.14, 95% CI: 1.05-1.23, p = 0.002; and HR: 1.10, 95% CI: 1.02-1.18, p = 0.010, respectively) [23]. Innate receptor signaling, intrinsic cell types, and systemic inflammation in PS may prompt untimely immunosenescence of the flowing CD8+ T cell compartment, which could have a clinical correlation for PS comorbidities, especially the inability to treat cancer. PS has an increased risk of developing several types of cancer. Also, IL-17A is generally linked to disease severity, while higher IL-6 levels have a strong association with PsA [24].

Impact of metabolic disorder in the treatment of psoriatic conditions

There are several factors to take into considerations before starting treatment in psoriatic diseases. PsA and severe PS have been associated with the use of adalimumab, tuberculosis, and young patients on ustekinumab. Chronic pulmonary diseases appear to drive the use of etanercept or ustekinumab over adalimumab. Etanercept is associated with a trend for prescription in patients with cardiovascular comorbidities, metabolic syndrome, and a history of cancer. However, patients with more cardiometabolic comorbidities are elderly populations. There are studies where secukinumab efficacy in elderly subjects was comparable to that of younger people. PASI 75 response was reached by 81.8% of elderly subjects and 79.4% of younger ones (69.3 years vs. 42.9 years, p = <0.0001). On the other hand, a prospective study showed that ustekinumab could decrease serum levels of adipocytokines (chemerin and resistin) in psoriatic patients, which can improve the metabolic profile of these patients and thus the severity of PS [25,26,27]. These are directly linked to a more severe form of the disease and a decrease in response to treatment. Psoriatic patients without MS after narrowband ultraviolet B ( NB-UVB) therapy showed a much less reduction of systemic biomarkers (IL-17, TNF-α, IL-6) in comparison to patients without MS. Significant PASI improvement was observed in psoriatic patients without MS compared to MS patients (p = <0.05) [28]. Similar results were observed with etanercept, where a BMI of ≥30 kg/m2 showed a decreasing effect on its efficacy [29]. On the other hand, pioglitazone and metformin have demonstrated improvement in the components of MS as well as PASI, erythema, scaling and induration (ESI), and physician global assessment (PGA) scores in pioglitazone (p = <0.05) [30]. The Charlson comorbidity index (CCI) was evaluated in a cohort study where a CCI comorbidity score of ≥2 was linked with poorer tumor necrosis factor inhibitor (TNFi) response in patients with PsA compared with patients without comorbidities (HR: 1.72, 95% CI: 1.26-2.37; p = 0.001). Additionally, patients with higher CCI comorbidity scores had higher disease activity measures and increased occurrence of psychiatric conditions [31]. Inverse PS is usually called a resistant type of skin lesion affecting the inguinal region, gluteal cleft, axillae, and external genitalia more frequently. It occurs more in people with high BMI and leads to further development of other conditions such as MS. Ustekinumab is suggested to be the drug with more effectiveness in this condition [32]. As we pointed before, comorbidities ought to be considered when choosing the best possible medication and biological treatment since they could result in negative consequences for metabolic parameters (Table 1) [25,26,27,33].

**Table 1:** Comorbidities affected by psoriasis medications WC: waist circumference; BMI: body mass index; NAFLD: non-alcoholic liver disease

Comorbidities	Medications and therapy	Side effects
NAFLD	Methotrexate	Liver fibrosis
Obesity	Acitretin	Dyslipidemia
Obesity, hypertension, older age	Cyclosporine	Nephrotoxicity
BMI score and greater WC	Phototherapy	Phototherapy-induced erythema

Obese patients (BMI: >40) impair clinical response to any systemic treatment for PS, especially anti-TNF-a drugs, which also have been reported to increase body weight. This is due to the belief that TNF antagonizes insulin. Bodyweight gain has not been registered in patients receiving secukinumab but, higher responses were showed in people who weighed <90 kg compared to those who weighed >90 kg after 12 weeks of treatment [29,33]. On the other hand, ustekinumab is prescribed according to weight ( >100 kg), showing better results in obese individuals, probably because, in contrast to anti-TNF-a inhibitors, ustekinumab does not increase BMI in PS patients [27,33]. Apremilast is a phosphodiesterase-4 inhibitor that can decrease weight, but no statistical relationship was found between weight loss by this drug and the severity of the disease [33]. As other studies have demonstrated before, treating comorbidities alongside PS is the best approach to decrease disease severity [30]. A faster and more effective decrease in PASI score in patients on oral simvastatin than in patients not on simvastatin was found in a case-control study after eight weeks of therapy (8.63 ±4.78 versus 5.34 ±3.59, p = < 0.01). Besides the control of dyslipidemia, which has proved to improve PS, the anti-inflammatory and immune-modulatory effects of statins influence in a variety of mechanisms of cholesterol level modulation, producing beneficial effects on psoriatic lesions [34]. Although methotrexate was associated with liver fibrosis in a patient with NAFLD, diabetes, obesity, and heavy alcohol intake, it was found to be safe in PsA patients without the risk of developing DM. HbA1c levels in PsA patients with MS were 5.7±0.9% before, and 5.9±0.9% after methotrexate therapy (p = 0.250) [33]. However, HbA1c levels in PsA patients without MS were 5.6±0.4 % and 5.7±0.5% before and after Methotrexate therapy, respectively p = 0.506) [35]. Since some pharmacological treatments may negatively affect cardio-metabolic comorbidities (CMDs), several studies have been done to help clinicians with the Metabolic and other comorbidities assessment in psoriatic patients [33,36]. Liver-type fatty acid-binding proteins (FABP) might be a predictor of clinical response to systemic therapy and CMDs risk assessment (p = <0.05) [37].

Impact of lifestyle modification in the treatment of psoriatic diseases

Good nutrition and exercise might play an important role in the treatment of psoriatic diseases. New evidence published in recent studies suggest that significant weight reduction in psoriatic patients treated with immunomodulators might contribute to a positive impact in the effectiveness of treatment [33]. In a randomized clinical trial, PASI 75 was studied with the use of biological agents; ustekinumab achieved the best PASI improvement in both dietary intervention and control groups (p = <0.001). Etanercept achieved the least PASI improvement score showing the highest mean weight gain (p = <0.001). At week 24, the mean (±SD) weight loss was 12.9 ±1.2 kg in the diet intervention group and -1.5 ±0.5 kg in the control group. The average improvement in mean PASI score was 84% for the diet group, and 69% for the control group. PASI 75 was achieved by 85.9% in the diet group, and 59.3% in the control group (p = <0.001). The mean (±SD) body surface area values at week 24 were 3.3 ±4.4% and 8.1 ±6.9% in the diet group and control group, respectively [38]. Psoriatic patients have an increased risk for myocardial infarction (MI) and stroke. In a randomized trial, low energy diet (LED) effect was measured on traditional cardiovascular risk factors and on endothelial function assessed by peripheral arterial tonometry (PAT) and selected plasma markers, showing a reduction of BMI, diastolic blood pressure, resting heart rate, total cholesterol, very-low-density lipoprotein cholesterol, triglycerides, glucose, and HbA1c in the interventional group. Also, reduction of one plasma marker related to endothelial type-1, (mean difference: 3.48 ng/ml, 95% CI: 1.50-5.46 ng/ml, p = 0.001). PASI appeared to improve more in the LED group compared to controls, although this did not reach statistical significance (p = 0.06) [39].

The western lifestyle seems to be the inflammatory process behind the increasing incidence of psoriatic diseases in the past few decades, especially in men due to their different eating behaviors compared to females. In a case-control study, it was observed that male psoriatic patients consumed a higher percentage of total and simple carbohydrates, total fat, PUFA, and n-6/n-3 PUFA ratio and cholesterol. While the consumption of protein, complex carbohydrates, MUFA, n-3 PUFA, and fiber was lower than in the control group, pointing to the idea of low MUFA leading to systematic inflammation and further development of PS. Low MUFA PASI score (r2 = 0.387, β = −0.635, t = −5.127, p = <0.001). MS presence was well predicted by higher PASI score (OR = 1.794; p = 0.002; 95% CI: 1.242-2.591) independently of low MUFA intake [40]. As mentioned before, chronic skin conditions may lead people to unhealthy lifestyles. Raising awareness of this type of condition is crucial. This type of intervention can improve the health-related quality of life (HRQoL) and avoid the development of psychological conditions in people with chronic inflammatory skin diseases [41]. The dietary intervention has to be one of the primary approaches in PS. It has been observed that psoriatic patients have essential mineral and vitamin deficiencies that may lead to systemic inflammation and worsening of the disease due to the production of reactive oxygen species. Gluten-free, ketogenic, fasting periods or vegetarian diets, and diets rich in omega-3 polyunsaturated fatty acids (ω-3 PUFA) are associated with an improvement of PS in clinical trials. On the other hand, Mediterranean diet (MD) (vegetables, fruits, whole grains and healthy fats. fish, poultry, beans and eggs, low meat and dairy intake), MUFA, and the most frequently consumed MUFA-rich dietary oils such as extra virgin olive oil (EVOO) are associated with lower level of CRP. It was found that a higher percentage of psoriatic patients have lower adherence to the MD [42]. In line with these literature, in a case-control study, a higher percentage of psoriatic patients had a lower/average adherence score compared to the control group (30.6% vs 4.8%, p = <0.001 and 51.7% vs 77.5%, p = 0.004, respectively) as assessed by the PREDIMED questionnaire (a validated fourteen-item questionnaire for the assessment of adherence to the Mediterranean diet). Also, there was a significant positive association among PASI score and BMI, FM (fat mass), WC, and PREDIMED score. In particular, psoriatic patients have the lowest values of Na/K ratio, FFM (fat-free mass), SMM (skeletal muscle mass), TBW (total body water), and higher values of FM. BMI, WC, and FM were significantly higher, and the PREDIMED score was lower than in the control group (p =<0.05) [43]. Contrary to these findings, in a controlled trial that measured the effect PUFA on cardiac autonomic function and vascular function in patients with psoriatic arthritis, blood pressure, central blood pressure, pulse wave velocity (PWV), did not change after supplementation with n-3 PUFA in the n-3 PUFA group but there was a trend towards an increase in RR (inverse of heart rate, varies from beat to beat) (p = 0.13) and decrease in heart rate (p = 0.12) in comparison with the control group. The group taking n-3 PUFA had a significant increase in the total content of n-3 PUFA (docosahexaenoic acid, EPA eicosapentaenoic acid, DPA docosapentaenoic acid) in granulocytes compared to the control group. There was also a significant decrease in the granulocyte content of arachidonic acid (AA) and linoleic acid (LA) in the n-3 PUFA-supplemented group compared to the control group. A significantly higher RR in the patients with the highest fish intake at baseline, which translates into a reduction of CVD [44]. Now more than before, understanding the basic science of weight management and the implementation of strategies to follow a healthy lifestyle should be the key and first-line treatment in many pathologies, including PS. Presently the rising number of researches involving the development of meaningful ways to achieve changes in weight and a further decrease of disease incidences and prevalence is booming and changing the way of practicing medicine today. In two separate studies, The Impact of intermittent fasting (Ramadan fasting) was measured in patients with PS and PsA, observing surprising results. A decrease in PASI score after the Ramadan fasting was noted (mean difference: −0.89 ±1.21, p = <0.0001). Apremilast (p = 0.0009), and phototherapy (p = 0.0015) were associated with a high PASI score before the Ramadan fasting. Similarly, the consumption of cyclosporine (p = <0.0001), IL-17 blockers (p = <0.0001), mammalian target of rapamycin or mTOR inhibitors (p = 0.0081), and TNF blockers (p = 0.0017) predicted a low PASI score after the Ramadan fasting. By contrast, narrow-band ultraviolet light B or NB-UVB (p = 0.0015) was associated with a high PASI score after Ramadan fasting. It is important to mention that Ramadan fasting could cause changes in sleep patterns, and it is well-known that sleep quality is closely related to the quality of life and several diseases, including PS [45]. After a month of intermittent fasting, CRP levels decreased from 14.08 ±4.65 to 12.16 ±4.46 (p = <0.0001), Bath Ankylosing Spondylitis Disease Activity Index (BASDAI) decreased from 2.83 ±1.03 to 2.08 ±0.67 (p = 0.0078), PASI decreased from 7.46 ±2.43 to 5.86 ±2.37 (p = <0.0001), and Disease Activity Index for Psoriatic Arthritis (DAPSA) decreased from 28.11 ±4.51 to 25.76 ±4.48 (p = <0.0001). Similarly, enthesitis improved after fasting, with Leeds Enthesitis Index (LEI) decreasing from 2.25 ±1.11 to 1.71 ±0.86 (p = <0.0001) and dactylitis severity score (DSS) decreasing from 9.92 ±2.93 to 8.54 ±2.79 (p = 0.0001) [46]. Weight management is related to a decrease in the severity of PS in overweight or obese individuals, proved by the improvement of PASI score in patients with a pooled mean difference of −2.49 95% CI: −3.90 to −1.08; p = 0.004) [47].

## Conclusions

A great extent of researches has been conducted on the subject showing significant evidence that the relationship between MS and PS is bidirectional, and are more than coincidental illnesses of each other. MS, as well as other comorbidities discussed here, and psoriasis have been hypothesized to be linked through inflammatory mechanisms. This paper endorses that hypothesis. MS has been found to have increased serum levels of cytokines in patients afflicted with the disease. These same proinflammatory mechanisms are responsible for causing psoriatic lesions and increasing PS severity. However, our paper showed that PS encompasses several factors including fatty acid, cytokines, adiponectin, CER abnormalities, among other immunological, biomolecular, and genetic factors, which may lead to further development of MS. Also, hypertension and gallstones diseases may play a significant part in the development of PS. This review endorses the view that nutritionists and specialists of preventive medicine should play a central role in the evaluation and management of psoriatic patients. We recommend the disease be primarily by focusing on the environmental and social factors instead of the genetic and immunologic pathways. Further studies are needed to explore the possibility of PS being secondary to the underlying way of a living dysfunction by the induction of psoriatic skin lesions in a specific patient population.
